# Advanced Light Source Analytical Techniques for Exploring the Biological Behavior and Fate of Nanomedicines

**DOI:** 10.1021/acscentsci.2c00680

**Published:** 2022-08-01

**Authors:** Mingjing Cao, Kai Zhang, Shuhan Zhang, Yaling Wang, Chunying Chen

**Affiliations:** ‡ CAS Key Laboratory for Biomedical Effects of Nanomedicines and Nanosafety & CAS Center for Excellence in Nanoscience, National Center for Nanoscience and Technology of China, Beijing 100190, China; † Beijing Synchrotron Radiation Facility, Institute of High Energy Physics, Chinese Academy of Sciences, Beijing 100049, China; § The GBA National Institute for Nanotechnology Innovation, Guangzhou 510700, China

## Abstract

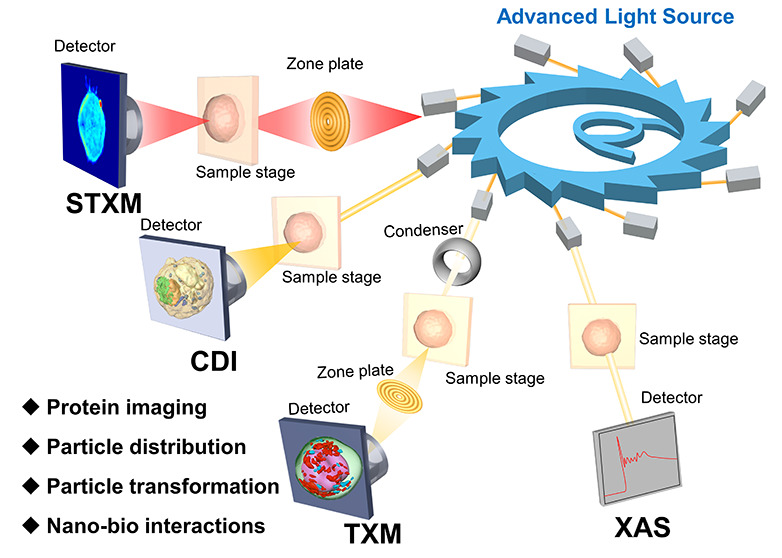

Exploration of the biological behavior and fate of nanoparticles, as affected by the nanomaterial–biology (nano–bio) interaction, has become progressively critical for guiding the rational design and optimization of nanomedicines to minimize adverse effects, support clinical translation, and aid in evaluation by regulatory agencies. Because of the complexity of the biological environment and the dynamic variations in the bioactivity of nanomedicines, *in-situ*, label-free analysis of the transport and transformation of nanomedicines has remained a challenge. Recent improvements in optics, detectors, and light sources have allowed the expansion of advanced light source (ALS) analytical technologies to dig into the underexplored behavior and fate of nanomedicines *in vivo*. It is increasingly important to further develop ALS-based analytical technologies with higher spatial and temporal resolution, multimodal data fusion, and intelligent prediction abilities to fully unlock the potential of nanomedicines. In this Outlook, we focus on several selected ALS analytical technologies, including imaging and spectroscopy, and provide an overview of the emerging opportunities for their applications in the exploration of the biological behavior and fate of nanomedicines. We also discuss the challenges and limitations faced by current approaches and tools and the expectations for the future development of advanced light sources and technologies. Improved ALS imaging and spectroscopy techniques will accelerate a profound understanding of the biological behavior of new nanomedicines. Such advancements are expected to inspire new insights into nanomedicine research and promote the development of ALS capabilities and methods more suitable for nanomedicine evaluation with the goal of clinical translation.

## Abstract 2

As label-free and *in-situ* analytical methods, advanced light source-based technologies, including microscopy and spectroscopy, hold great potential for exploring the biological behavior and fate of nanomedicines.

## 1 Introduction

After decades of nanotechnology development, innovative nanomedicines show outstanding performance in various biomedical fields, such as diagnosis, drug delivery, and therapy.^1−3^ The unique physical and chemical properties of nanomaterials present a double-edged sword in the current clinical applications of nanomedicines.^4−9^ While these versatile formulations have greatly improved the safety and efficacy of traditional medicines, nanomedicines face obstacles in manufacturing, preclinical characterization, and clinical translation.^10−14^ The intrinsic physicochemical properties (valence state, size, charge, etc.) of nanomedicines not only facilitate their medical efficacy but also affect the final destiny of nanomedicine in the body.^15−20^ For example, the surface protein corona, which forms *in vivo* or may be fabricated prior to administration, alters the inherent activities and distribution of nanomedicines at interfaces, barriers, and biological structures by affecting the bioidentity of nanomedicines.^21−26^ Meanwhile, the transformation of nanomedicines within biological systems induces variations in the surface properties, structures, and functions.^27−31^

One aspect of nanomedicines that remains poorly understood is the true physiochemical behavior of nanostructures inside the dynamic biological environment. The biological behavior and fate of nanomedicines,^30,32−35^ and the information underlying nanomaterial–biology (nano–bio) interactions,^36−41^ spatiotemporal relationships among networks of nanoparticles (NPs),^42−45^ their metabolic products,^28,46−49^ and cell components must be defined. Understanding these relationships will allow us to grasp how nanomedicines interact with their surrounding biological environments and how variations in nanoformulations manifest protein corona characteristics as well as cellular responses, such as oxidative stress, genetic damage, and toxicity. However, *in-situ* and label-free analysis of the interactions between nanomedicines and biological systems with high resolution and sensitivity remains a challenge. Current imaging techniques including electron microscopy (e.g., transmission electron microscopy and scanning electron microscopy), optical microscopy (fluorescence microscopy, etc.), and positron emission tomography/single photon emission computed tomography (PET/SPECT) have contributed tremendously to analyzing the behavior of nanomedicines in biological environments. Though electron microscopy captures images with high resolution, it is extremely difficult to image the internal structure within intact cells or tissues, observe *in situ*, and analyze quantitatively. Fluorescence microscopy, particularly super-resolution microscopy, can reveal the dynamic behavior of nanomedicines with high resolution, despite somewhat slow progress and the lack of versatile fluorescence probes. PET/SPECT enables imaging of small animals and humans but requires the label of nanomaterials with radioactive tracers. Lately, X-ray, especially advanced light source (ALS)-based technology, has been emerging as a powerful analytical tool to understand the nano–bio interaction. The X-rays generated from ALS facilities have high brilliance, collimation, and a broad energy range (UV to several tens of keV); they can penetrate deeply inside samples and interact with matter to produce absorption, phase, and fluorescence signals. ALS techniques have multiple advantages: label-free, *in-situ*, high resolution, quantitative analysis, high penetration depth, and simple sample preparation, which enable the study of the biological behavior and fate of nanomedicines in cells or tissues with native or near-native states.^50−53^ From the molecular to the tissue levels, current X-ray methods can provide information on the chemical environment, agglomeration, and spatial distribution of NPs.

In this Outlook, we introduce a selection of ALS imaging and spectroscopic technologies with which it is possible to obtain the two-dimensional (2D) or three-dimensional (3D) distribution and the transformation of nanomedicines, as well as the morphology of affected cells. We also provide typical examples of the applications of these techniques to understand the biological behavior and fate of nanomedicines, and summarize key information that can aid researchers in the design study and choice of various beamlines. It is our hope that this Outlook will broaden the applications of ALS analytical methods in nanoscience.

## 2 Overview of ALS Imaging and Spectroscopic Technologies

### 2.1 Basic Theory

In this section, we present a detailed discussion about the basic principles of selected X-ray microscopy and spectroscopy, including full-field transmission X-ray microscopy (TXM), scanning transmission X-ray microscopy (STXM), coherent diffraction imaging (CDI), and X-ray absorption spectroscopy (XAS), which have been developed to provide two- or three-dimensional (2D or 3D) insights into morphological information on nanomedicines, tissues, cells, or organelles with the resolution of tens of nanometers, and the chemical forms of nanomedicines.

**Figure 1:**
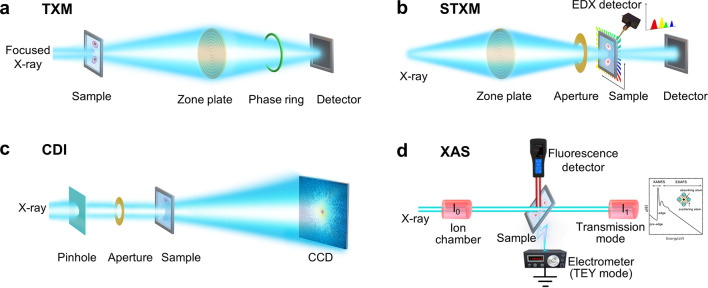
Schematic illustration of four major ALS imaging and spectroscopic technologies. (a) Transmission X-ray microscopy (TXM), (b) scanning transmission X-ray microscopy (STXM), (c) coherent diffraction imaging (CDI), and (d) X-ray absorption spectroscopy (XAS).

TXM, illustrated in Figure 1a, is based on the principle of projecting a magnified image of the sample obtained with a hollow cone illumination onto a detector.^54^ It is difficult to manufacture the zone plate for the hard X-ray region, so the TXM system cannot operate with X-rays higher than 15 keV. TXM is rapidly gaining popularity with instruments operating in the soft X-ray (180 eV–2 keV) and hard X-ray (5–15 keV) ranges. For soft X-ray TXM, because of the limited depth of field (DOF), only nanoscale structures of small-sized and thin biological samples are suitable for study in the absorption image mode. Meanwhile, hard X-ray TXM can image large-sized and thick samples in absorption image mode or Zernike phase-contrast image mode. The typical spatial resolution of the TXM system is about 10–100 nm in the soft X-ray region and 30–150 nm for hard X-rays. Moreover, X-ray computed tomography (CT) measurements can be achieved by reconstructing 2D projections of a rotating sample into a 3D image, thereby providing inner structure and morphology information about the sample.

In STXM (Figure 1b),^55,56^ the X-ray is focused by a combined KB mirror or zone plate onto a small spot containing the sample. A proportional counter collects the transmitted X-rays, and the images are built pixel by pixel. The spatial resolution of STXM is determined by the focused size of the X-ray beam and can reach 10 nm. Two-dimensional elemental and chemical distribution of the sample can be simultaneously obtained using multiple detectors (e.g., XRD, XRF) during the point-by-point scan. Moreover, STXM can generate near-edge X-ray absorption fine structure (NEXAFS) spectra for each pixel when coupled with the spectroscopy technique. By combining STXM-NEXAFS with CT techniques, it is possible to construct a 3D structure that includes the distribution of different chemical species and the valence states of atoms.

CDI (Figure 1c) using coherent third- and, especially, fourth-generation light sources is a lensless imaging method. A specimen is irradiated by X-rays with high-spatial coherence, and the diffraction pattern is collected with an area detector to form images from scattered light through advanced phase-retrieval algorithms.^57^ The CDI resolution is determined only by the largest scattering angle. Thus, compared with TXM and STXM, the key advantage of CDI techniques is that ultrahigh spatial resolution (below 10 nm, theoretical resolution may down to atomic level) can be achieved by avoiding the use of lenses. The other advantage of CDI is that it can also take advantage of the phase contrast between the intrinsic densities in biological specimens, thereby enabling quantitative imaging of the entire structures of cells and cellular organelles with natural contrast and without sectioning. Thus, CDI holds great potential for 2D and 3D analysis with applications in cells and organelles.

XAS (Figure 1d), encompassing the techniques of X-ray absorption near-edge structure (XANES) and extended X-ray absorption fine structure (EXAFS), is an analytical technique important to biological research. XAS is based on the principle whereby an absorbed photon interacts with an electron in the field of an incident X-ray to generate a time-dependent acceleration. The electron then is activated from a core-orbital to an unoccupied bound or continuum state with an intensity given by Fermi’s Golden Rule.^58^ By tuning the energy of a monochromatized beam to the binding energy of an element of interest in the sample, the absorption coefficient spectrum of that element can be measured in the ion chamber. XANES yields a direct measurement of the valence state, while EXAFS can quantitatively analyze the atomic structure details. The energy resolution in the XAS determined by the monochromators should be high for the XANES (Δ ≤ 0.2 eV) and can be low for the EXAFS (Δ ≈ 6 eV).^59,60^ XAS is usually included in the above-mentioned methods, which is promising for 2D or 3D element-specific imaging.

### 2.2 Comparison of the Major X-ray Analytical Methods in the Study of Nanomedicines

As presented in section 2.1, for X-ray imaging and chemical analysis of biological samples, each method has its advantages and limitations. Table 1 lists the performance of these techniques and some selected synchrotron facilities where relevant equipment is available, to provide a quick guide for method selection.^61−63^ Progress in X-ray imaging technologies includes the label-free, intact, high-resolution features to study the morphology of organelles and cells in biological specimens,^64−69^ especially 3D tomography of cell/organelle morphology, structure, quantity, and near-native distribution of nanomedicines at the resolution of tens of nanometers. The technical superiority of soft X-ray TXM/STXM is the capability to visualize hydrated cells with high resolution. Superhigh resolution can be achieved by the CDI technique. Three-dimensional imaging of a large and thin cell benefits from soft X-ray TXM, while STXM is not suitable because of the low scanning rate and impermeability. The major advantage of STXM is that it provides chemical information combined with spectroscopy. XAS can be used separately (bulk XAS) or combined with STXM (*in-situ* XAS) to provide overall composition information on an entire biological sample or specific regions of interest *in situ*. Samples in solution, frozen solution, solid, gas, crystalline, or amorphous are amenable to XAS measurements, which makes it extraordinarily suitable to analyze nanomaterials in biological specimens. These microscopes and spectroscopes are applicable to most biological research fields, such as the toxicity of nanomedicines, vaccine efficiency, virus infection, etc.

**Table 1:** Performance of Different ALS Analytical Methods^a^

							sample processing method		
technique	energy (keV)	working principle	resolution (nm)	2D sample size (μm × μm)	sample thickness (μm)	morphology/element mapping/chemical information	cell	tissue	synchrotron facilities
TXM	hard X-ray: 5–15	absorption/phase/K edge/XANES	≥30	<65	<65	yes/yes/yes	chemical fixation	chemical fixation, section	SLAC; BSRF; ESRF; APS; BESSY
	soft X-ray: <2	absorption/K edge/XANES	≥10	<10	<10	yes/yes/yes	cryogenic freezing	not applicable	ALS; BESSYII; NSLS; Elettra
STXM	hard X-ray: 5–20	absorption/XANES/fluorescence (XRF)	≥30	no limit	<65	yes/yes/yes	chemical fixation	chemical fixation, section	BNL; BESSY; SSRF
	soft X-ray: <2	absorption/XANES/fluorescence (XRF)	≥10	no limit	<10	yes/yes/yes	cryogenic freezing	not applicable	ALS; CLS; BESSYII; SSRF; SSRL; SLS
CDI	hard X-ray: 5–15	diffraction	≥1	no limit	<50	yes/yes/no	cryogenic freezing	chemical fixation, cryogenic freezing	LCLS; SLS; ESRF
	soft X-ray: <2	diffraction	≥1	no limit	<10	yes/yes/no	cryogenic freezing	not applicable	ALS; CLS; BESSYII; SSRF
XAS	hard X-ray: 5–25	XANES/EXAFS; combined with STXM/TXM	≥m-mm	no limit	<65	no/no/yes	bulk XAS: lyophilized, pressed to be a flat and uniform pellet		Spring-8; BSRF; SSRF; ESRF; APS; SLAC
	soft X-ray: <2	XANES/EXAFS; combined with STXM/TXM	≥m-mm	no limit	<10	no/no/yes	*in-situ* XAS: same sample preparation to the method of the corresponding imaging		ALS; BESSYII; NSLS; SLS; CLS; Elettra

a Note: Full names of the synchrotron facilities: Stanford Linear Accelerator Center (SLAC), Beijing Synchrotron Radiation Facility (BSRF), Shanghai Synchrotron Radiation Facility (SSRF), European Synchrotron Radiation Facility (ESRF), Advanced Photon Source (APS), BESSY Accelerator (BESSY), BESSYII Accelerator (BESSYII), Advanced Light Source (ALS), National Synchrotron Light Source (NSLS), Elettra Synchrotron Light Source (Elettra), Brookhaven National Laboratory (BNL), Canadian Light Source (CLS), Linac Coherent Light Source (LCLS), Swiss Light Source (SLS), Stanford Synchrotron Radiation Lightsource (SSRL), Super Photon Ring-8 GeV (SPring-8).

## 3 Visualization of Nano–bio Interactions from the Subcellular to Organ Levels Using ALS Imaging

### 3.1 Three-Dimensional Intracellular Distribution of Nanomedicines and Tomography Imaging of Cell Morphology with Soft X-ray Microscopy

From the perspective of biology, imaging natural cell structures without heavy metal staining to enhance contrast will provide the most faithful information regarding nano–bio interactions. In particular, soft X-ray TXM/STXM/CDI has the obvious advantages of visualization and quantification of volumes, surfaces, interfaces, and structural connectivity between organelles in hydrated cells through the absorption or phase-contrast techniques.^64,70,71^ Furthermore, to obtain a more quasi-natural state of the cell and minimize the radiation damage from soft X-rays, the cryogenic method is integrated and has opened a new era for 3D imaging.

**Figure 2:**
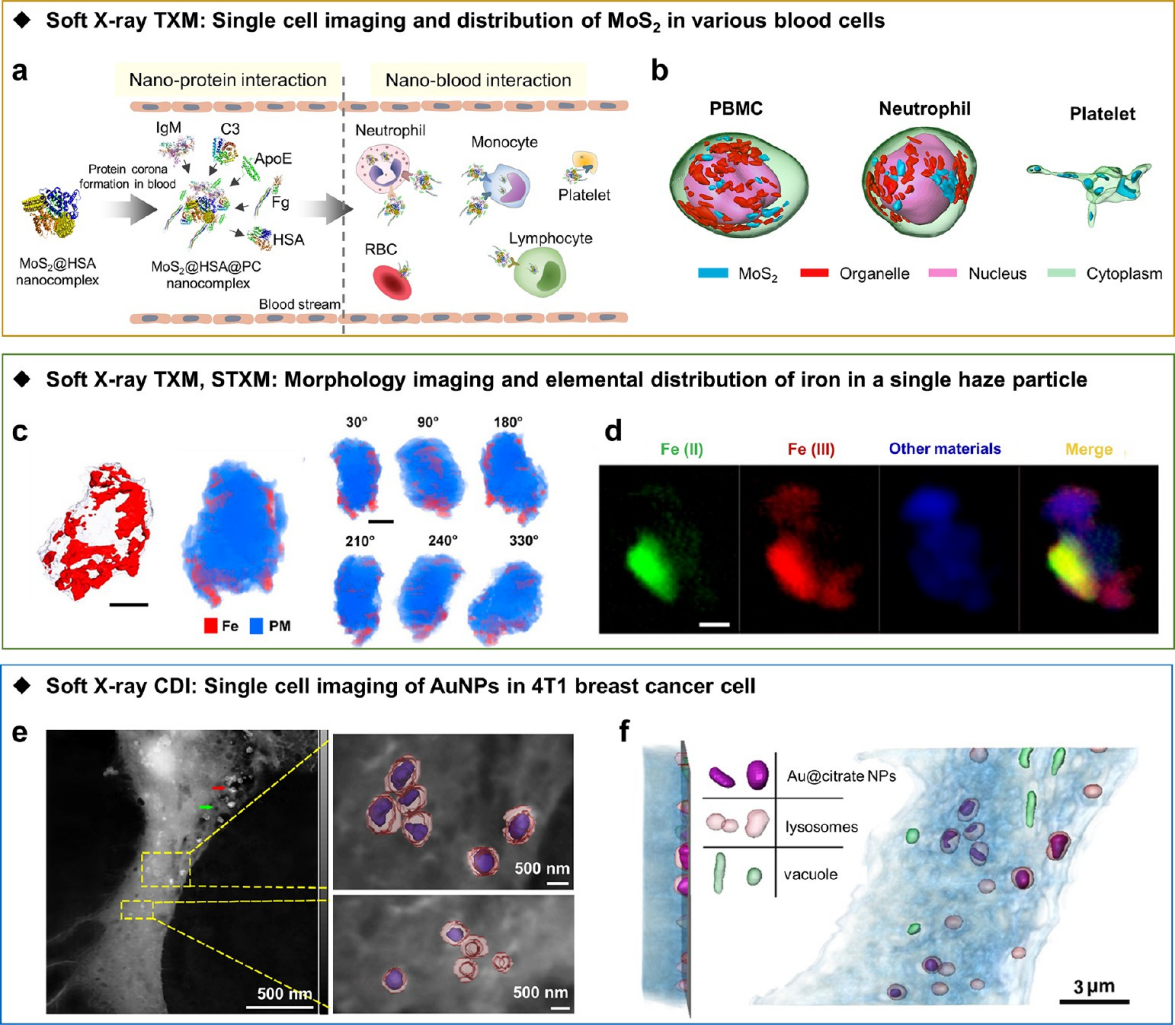
3D intracellular localization of nanoparticles with soft X-ray imaging. (a) Schematic illustration of the interactions of MoS_2_@HSA nanocomplexes with proteins and blood cells; (b) 3D reconstructed images of MoS_2_ in peripheral blood mononuclear cells (PBMCs), neutrophils, and platelets from cryo-soft TXM. (c) Spatial distribution of iron elements (red) in a single haze particle shown in 3D TXM tomographic images (scale bar: 1 μm); PM: particulate matter; (d) 2D distribution of ferrous and ferric irons, as determined by STXM coupled with NEXAFS (scale bar: 500 nm). (e) 3D segmentation of lysosomes (pink) and AuNPs (violet) from the 2D ptychography image (left) of a 4T1 cell; (f) 3D volume rendering of the 4T1 cell from YZ (left) and XY (right) plane showing the distribution of AuNPs in lysosomes. Panels a and b adapted with permission from ref (30). Copyright 2021 Springer Nature. Panels c and d reproduced with permission from ref (80). Copyright 2020 American Chemical Society. Panels e and f adapted with permission from ref (85). Copyright 2021 American Chemical Society

In 2004, the 3D image of an entire yeast cell imaged by cryo-soft TXM was achieved at the resolution of 60 nm.^67^ Later, additional biological structures from different organisms and cell types (*Candida utilis*, *Candida albicans*, erythrocytes, human stem cells, Vero cells, etc.) were imaged using soft X-ray TXM.^72,73^ Soft X-ray tomography facilitates the visualization of nano–bio interactions by providing 3D spatial information on cellular interactions and the quantitative distribution of NPs.^30,74,75^ For example, to understand the influence of the blood protein-derived corona on the *in-vivo* behavior of NPs, Chen et al. designed a series of experiments to investigate the *in-vivo* transport, transformation, and bioavailability of MoS_2_ nanomaterials after their intravenous injection.^30^ Three-dimensional reconstructed images with a spatial resolution of 30 nm obtained from unstained cryo-soft-TXM revealed the intracellular localization of MoS_2_ in various blood cells isolated from anticoagulated blood (Figure 2a,b). In addition to inorganic NPs, an element with a high atomic number (iodine) aided in the visualization of intracellular nanostructure formation of self-assembled organic NPs and the reconstruction of the 3D distribution of assembled NPs in HeLa cells, according to the linear absorption coefficient of iodine labeled NPs.^75^

Soft X-ray STXM is complementary to TXM in capabilities. The former has been applied to image bacteria, yeast cells, macrophages, and cancer cells.^76−79^ With the combination of soft X-ray STXM and the equally sloped tomography (EST) algorithm, Jiang et al. examined the quantitative 3D subcellular distribution of Gd@C_82_(OH)_22_, a promising antitumor nanomedicine, within a macrophage.^77^ Reconstructed 3D images demonstrated the location of aggregated Gd@C_82_(OH)_22_ in cytoplasmic phagocytic vesicles and the absence of the NPs in other organelles (e.g., nuclei). Moreover, the internalization of Fe_3_O_4_–SiO_2_ NPs in Hela cells was investigated with 3D tomography performed with a new generalized Fourier iterative reconstruction algorithm of the STXM projections at the X-ray energy of the Fe L-edge.^79^ The lower photon fluxes in soft X-ray STXM, when compared to TXM tomography, reduce the radiation damage of the sample. However, the data acquisition rate is slow since the samples are raster scanned in STXM imaging, and the images are built pixel by pixel. The major advantage of STXM tomography is the capability to provide the chemical structures of elements in the specimen. For example, the spatial distribution of chemical species of iron in single haze particles was investigated with soft X-ray STXM and TXM.^80^ Three-dimensional tomographic images reconstructed from TXM projections showed that aggregated iron atoms were mainly present close to the surface of the particulates (Figure 2c). The *in*-*situ* distribution of iron chemical forms, as determined with STXM coupled with NEXAFS based on PCA analysis and stack data fitting, suggested a broad distribution of the ferric form and the main location of ferrous ions within the particle (Figure 2d). The ferrous form of iron can catalyze the generation of hydroxyl radicals, which suggests the potential to damage respiratory and cardiovascular systems.

To achieve a higher spatial resolution (<10 nm), CDI can image thick biological samples with coherent X-rays and reconstruct data with an iterative algorithm under a low radiation dose. To date, the CDI technique has been widely applied to quantitatively determine the structures of yeast spores,^68^ yeast cells,^81,82^ green algae,^83^ bacteria,^84^ various mammalian cells,^79,85^ and organelles^86^ in 2D or 3D, holding great potential for bioimaging at the single-cell level. Furthermore, CDI is a powerful tool, capable of revealing nano–bio interactions with high resolution and contrast. By combining soft X-ray 3D ptychography CDI and the EST algorithm, the intracellular distribution and transport behavior of Au@citrate NPs in relatively large and flat murine breast cancer cells (4T1, size: 72.13 μm × 38.54 μm × 1.4 μm) were quantitatively evaluated (Figure 2e,f).^85^ Organelles, including the nucleus, intracellular vesicles, multivesicular bodies (MVBs), lysosomes, and mitochondria, were clearly discriminated. The majority of the Au@citrate NP aggregates were present in MVBs and lysosomes, which was verified by confocal fluorescence microscopy and electron microscopy. The authors also found that the lysosomes encasing AuNPs of different shapes were ∼2 times larger than those without AuNPs. One shortcoming of the study was that the natural state of the cells was not maintained due to fixation and dehydration procedures.

**Figure 3:**
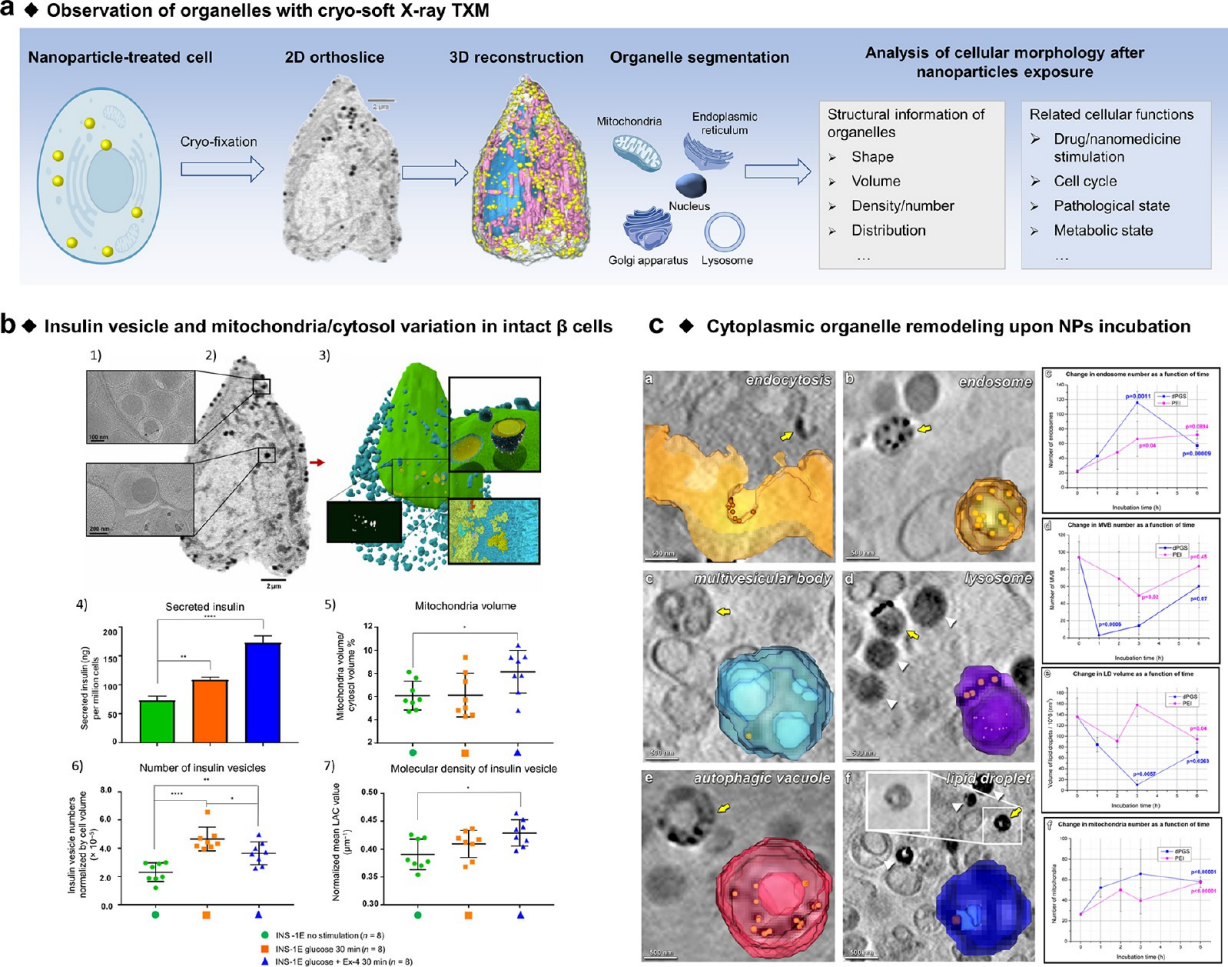
Three-dimensional investigation of cellular structures with cryo-soft TXM. (a) Schematic illustration of the brief workflow of *in-situ* imaging the intact cell by cryo-soft TXM. Images in “2D orthoslice” and “3D reconstruction” are reproduced with permission from ref (70). Copyright 2020 American Association for the Advancement of Science. (b) Three-dimensional spatial rearrangements of insulin vesicles and cytosol variations in intact β cells after glucose and the drug exendin-4 (Ex-4) stimulation. (1) Representative electron tomography image of an INS-1E rat insulinoma cell showing different subcellular environments in the margin and center of the cell as indicated. (2) Representative 2D orthoslice portraying whole-cell architecture. (3) Three-dimensional molecular model of a single β cell. Nucleus (green); insulin vesicles (blue); core of insulin vesicles (yellow); atomic details of protein packing (zoom views); a rendering of the segmented vesicle mask (black widow). (4) Insulin secretion with cells measured by enzyme-link immunosorbent assay (ELISA). Plot of (5) mitochondria/cytosol volume ratios, (6) number of insulin vesicles, and (7) mean insulin vesicle linear absorption coefficient (LAC) value. Reproduced with permission from ref (70). Copyright 2020 American Association for the Advancement of Science. (c) Cytoplasmic changes affected by AuNPs exposure. Left images: endocytic uptake of dPGS-AuNPs in A549 cells investigated via 3D rendering of the cellular structure. AuNPs are rendered in gold color. Right plots: changes in the number of endosomes, MVB, mitochondria, and lipid droplet volume as a function of time after incubation with dPGS-AuNPs and PEI-AuNPs. Adapted with permission from ref (89). Copyright 2020 American Chemical Society.

To investigate the impact of nano–bio interactions and characterize cell organelle networks’ structure and functions of whole and unstained cells with near-native states, the cryogenic method can be integrated with X-ray microscopy.^70,74,87−89^ The subcellular morphology including the number, volume, density and integrity of organelles under multiple cellular physiological or pathological conditions, such as infection, disease progression and nanoparticle/nanomedicine treatment, can be visualized in hydrated cells (Figure 3a). For example, White et al. used soft X-ray tomography to visualize the subcellular rearrangements and insulin particle secretion in intact β cells under different glucose-stimulated conditions (Figure 3b).^70^ Rapid alterations in the component and density of insulin, increased mitochondrial volume, and closer contact of insulin vesicles to mitochondria were induced by glucose stimulation, which was prolonged by the costimulation of glucose with drug exendin-4 (Ex-4). McNally et al. demonstrated changes in the number of cytoplasmic organelles (mitochondria, endosomes, lipid droplets, multivesicular bodies) within human lung epithelial cells (A549) induced by the uptake of dendritic polyglycerol sulfate (dPGS)/polyethylenimine (PEI)-coated AuNPs by cryo-soft TXM.^89^ As shown in Figure 3c, after incubation with the AuNPs, different organelles (endosomes, lysosomes, MVBs, autophagosomes, lipid droplets) internalized these NPs. The researchers also focused on cytoplasmic remodeling after AuNPs exposure. The number of lipid droplets (LD) and multivesicular bodies (MVB) decreased, and later increased, which were opposite effects compared to the mitochondria (M) and endosomes (E) at the same time point. The remodeling and rearrangement of subcellular architectures induced by nano–bio interactions imply the disturbance to cell functions.

### 3.2 Three-Dimensional Imaging of Intracellular Nanomedicines via Hard X-ray Microscopy

**Figure 4:**
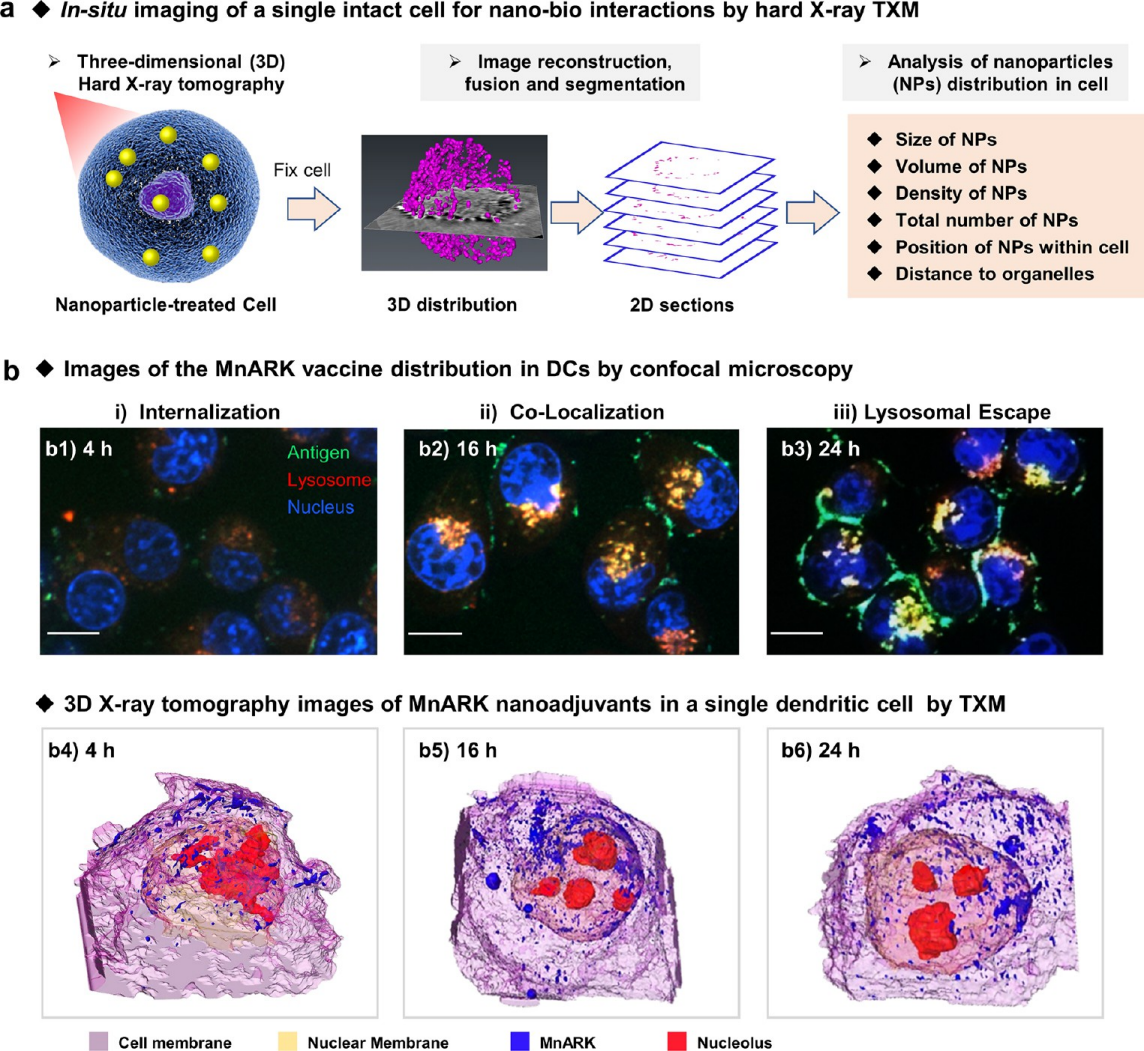
Three-dimensional visualization of NPs in cells by hard X-ray TXM. (a) Schematic illustration of the strategy of *in-situ* imaging the intact cell in the nano–bio interaction by TXM. The 3D distribution and 2D section images are adapted and reproduced with permission from ref (28). Copyright 2015 American Chemical Society. (b) Intracellular localization imaging of RBD and MnARK with confocal fluorescence microscopy (b1–b3) and X-ray tomography (b4–b6). Adapted with permission from ref (91). Copyright 2021 Elsevier.

Soft X-rays, especially the X-rays in the “water window”, are well-suited to imaging the structures of unstained, hydrated, and native cells with high resolution. However, only small-sized and thin biological samples are acceptable for soft X-ray imaging due to the limited depth-of-focus of soft X-rays (<10 μm). For large-sized and thick cell samples (majority of eukaryotes), hard X-ray microscopy has obvious advantages in 3D cell imaging,^28,90−92^ enabling the *in-situ* analysis of the intracellular behavior of nanomedicine including the distribution, volume, density, total number and size of NPs, the position of NPs within the cell, and the distance to a specific organelle (Figure 4a) in an intact cell. For example, exploiting hard X-ray TXM, Chen et al. described the intracellular uptake and exocytosis of 20 nm Ag NPs in THP-1 cell (size: 15 μm × 15 μm × 15 μm).^28^ Recently, the same group designed a SARS-CoV-2 RBD vaccine by using albumin-templated nanosized Mn adjuvant (MnARK) NPs as both adjuvants and cargos, with the RBD antigen aiding the uptake of the NPs by dendritic cells (DCs).^91^ TXM acquired with a Zernike phase-contrast imaging model revealed that MnARK can be internalized by DCs and accumulate in a time-dependent model (Figure 4b), which follows the same pattern as the antigen. Thus, the electrostatic-driven formation of RBD antigen corona on the MnARK surface promoted the internalization of RBD. Moreover, Gu et al. investigated the translocation, degradation, and toxicology of MoS_2_ nanosheets (NSs) by combining traditional analytical methods with ALS techniques;^92^ integrating TXM images with contrast signals from X-ray absorption revealed the presence of NSs in the lysosomes of hepatoma cells. The NPs decreased significantly after exocytosis for 12 h. Because of the excellent penetration capability of hard X-rays, it may be needed to stain cells or label cells with X-ray signal probes to investigate the cellular structure.

### 3.3 Imaging of Intracellular Proteins with X-ray Signal Probes

**Figure 5:**
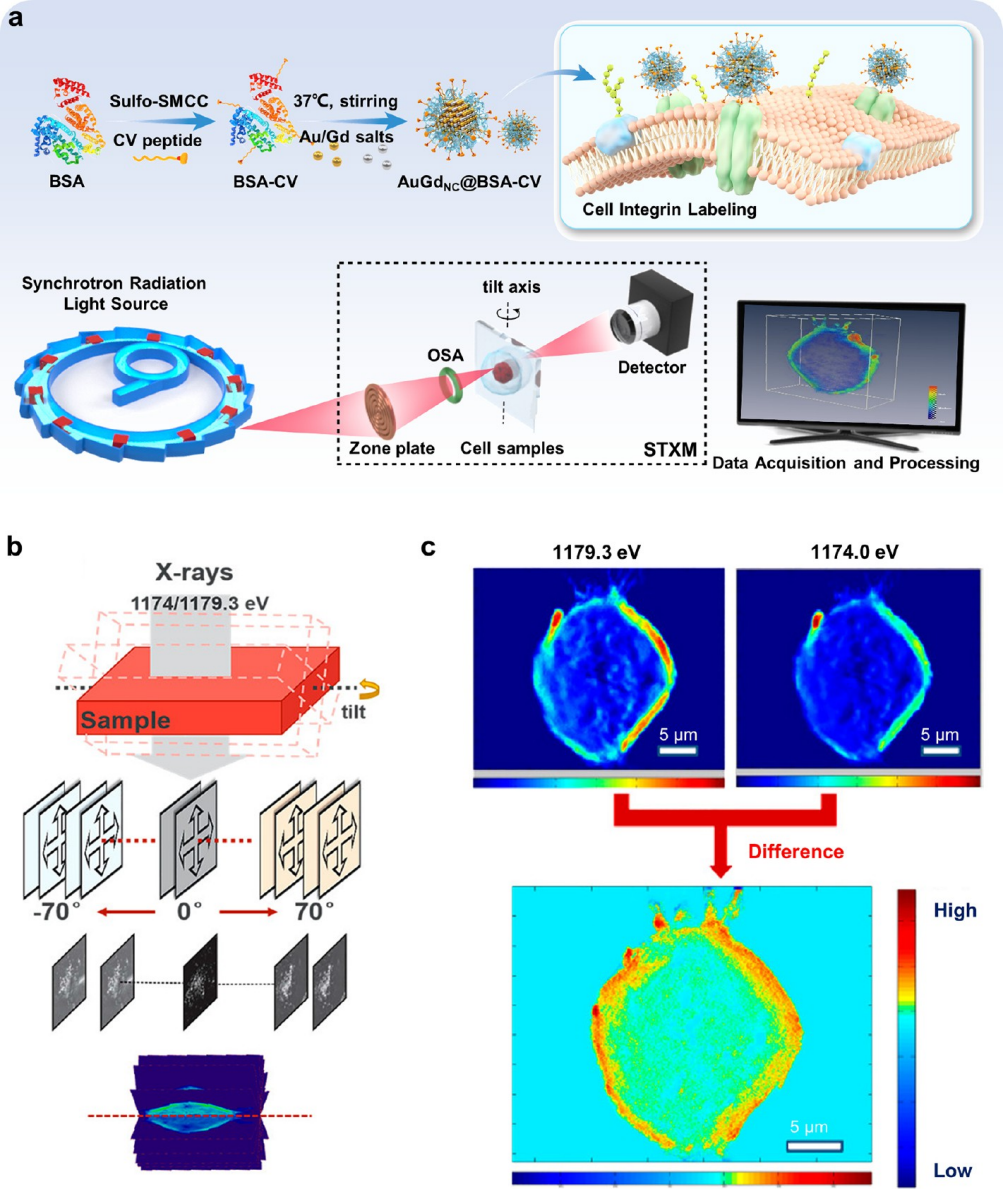
Visualization of integrins on the cell membrane with X-ray signal probes. (a) Schematic illustration of the X-ray-sensitive AuGd nanoprobe preparation and application for integrin-targeted 3D imaging. (b) Image acquisition process of dual-energy STXM. (c) Two projections at a 0° tilt angle, acquired by dual-energy STXM at energies above (1179.3 eV) and below (1174.0 eV) the absorption edge of the Gd element, and the reconstruction using the EST algorithm. Reproduced with permission from ref (93). Copyright 2021 American Chemical Society.

**Figure 6:**
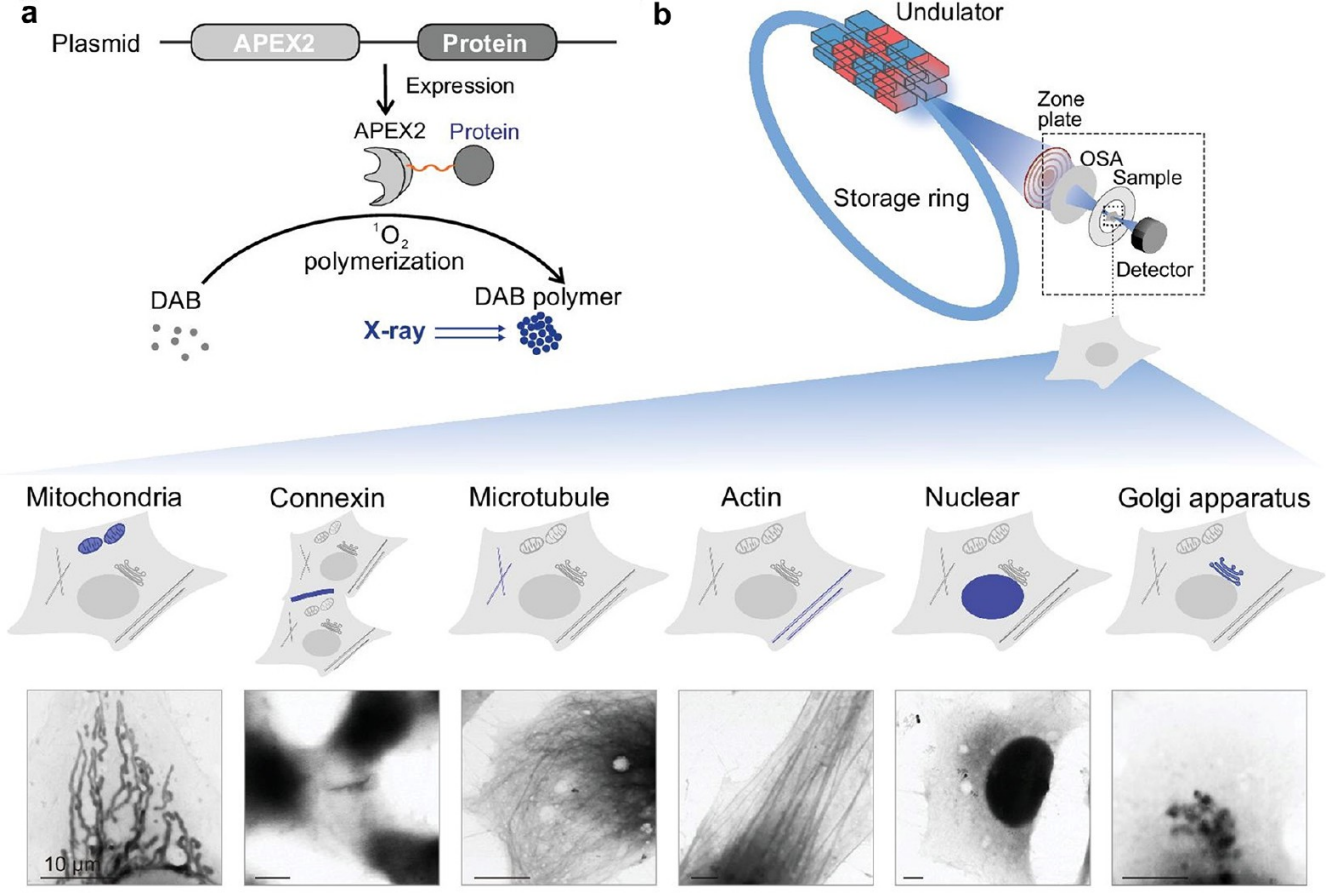
Investigation of specific proteins in organelles with genetically encoded peroxidases as X-ray probes. (a) Schematic illustration of genetically encoded peroxidases (APEX2) as probes for protein localization with STXM. (b) STXM images of cellular proteins and specific amino acid sequences: cytochrome c oxidase subunit 4 (mitochondria), connexin-43, α-tubulin, β-actin, nuclear localization sequence, and galactosyltransferase (Golgi apparatus). Reproduced with permission from ref (94). Copyright 2020 Oxford Academic.

The distribution and expression of proteins are of great consequence to the *in-vivo* behavior and fate of nanomedicine. Because of the chemical similarity of proteins, X-ray imaging methods are insufficient to image specific proteins directly with high resolution. The development of X-ray-enhanced nanoprobes can aid in the functional study of nano–bio interactions. Thus, probes with X-ray absorption, fluorescence, and phase signals have been developed to visualize intracellular proteins, including immune Au NPs or lanthanide metal tags.^93−95^ Wang et al. designed an AuGd nanoprobe conjugated with an integrin-targeting peptide to visualize the 3D distribution of integrin on the human erythroleukemia cell membrane (Figure 5).^93^ Using an alternative method to labeling proteins with targeted X-ray-sensitive probes, Fan et al. encoded peroxidases (APEX2) to label specific proteins in organelles; the recombinant protein catalyzed the formation of DAB precipitates to form DAB polymers *in situ* (which exhibit stronger X-ray absorption in the water window) enabling the imaging of these proteins (Figure 6).^94^ Dual-color imaging of cells was achieved by introducing two genetically encoded peroxidases and another substrate containing cobalt, with absorption energies different from those of DAB. Similarly, Miller et al. reported an encoded fusion tag with high lanthanide metal affinity in outer membrane protein A (OmpA).^95^ The authors thus visualized the 3D distribution of OmpA in *E. coli* with X-ray fluorescence microscopy at nanoscale resolution. With the development of next-generation ALS, X-ray nanoprobes are expected to open new avenues in X-ray microscopy, enabling high-resolution imaging of whole-cell morphology and functional investigation of the nano–bio interaction.

### 3.4 *In-Vivo* Spatial Distribution of Nanomedicines by ALS Microscopy

**Figure 7:**
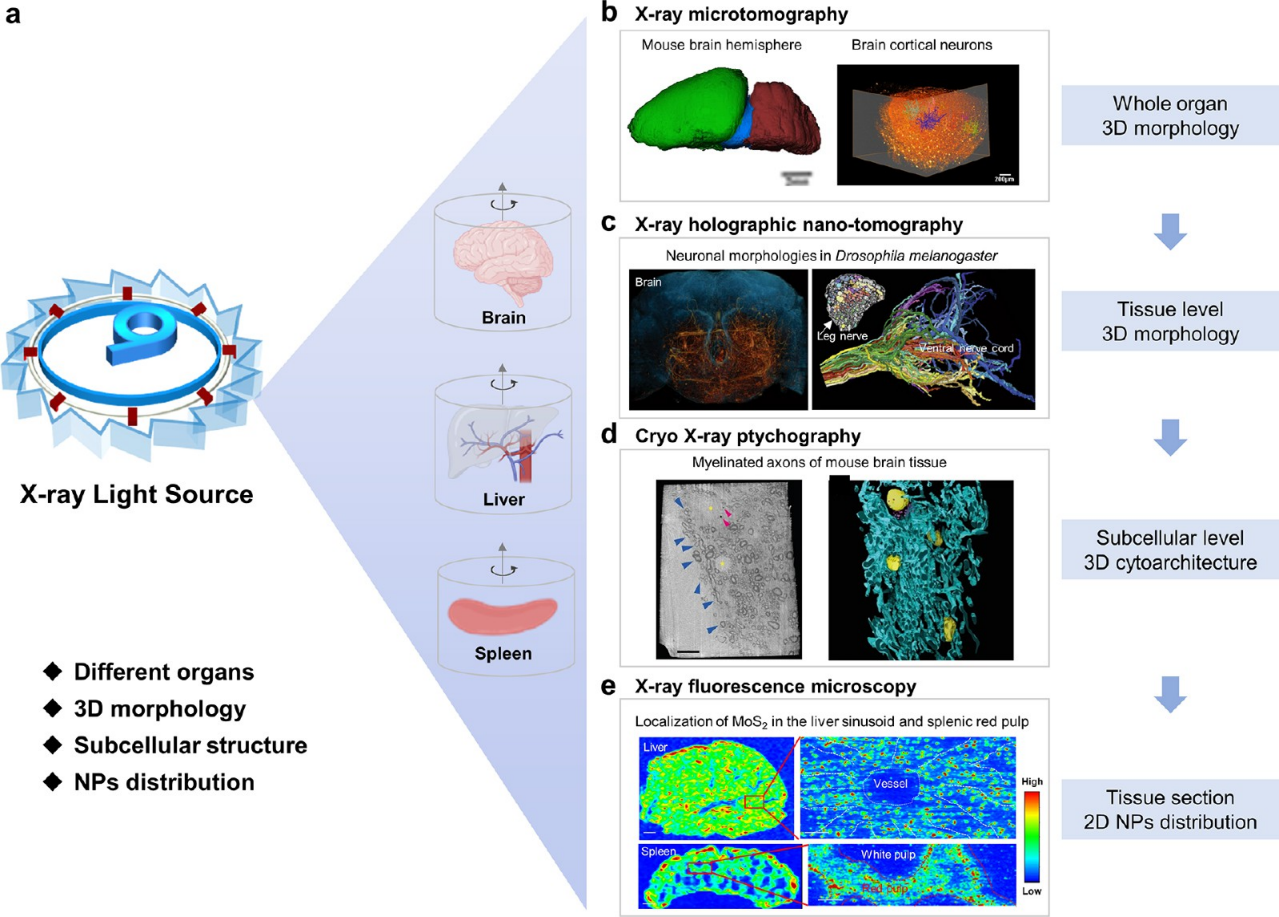
Different X-ray microimaging techniques provide detailed 2D/3D information about tissue architecture and distribution of NPs in tissues with high resolution at the microscale from whole tissue to subcellular levels. (a) The X-ray from ALS can be used to irradiate different organ tissue and nanoparticles. Images of brain, liver and spleen tissues are created with BioRender.com. (b) Three-dimensional morphology of a whole mouse brain and cortical neurons with X-ray microtomography. Adapted with permission from ref (96). Copyright 2018 Springer Nature. (c) Neuronal morphologies in *Drosophila* brain (left), leg, and ventral nerve cord (right) imaged by X-ray holographic nanotomography. Left: Three-dimensional volume rendering of the central fly brain. The brain outline (blue); neurons (orange). Right: Main image is the automatically segmented neurons in the *Drosophila* ventral nerve cord, while the inset is a cross-section of the main leg nerve, with colors showing different neuron types. Reproduced with permission from ref (97). Copyright 2020 Springer Nature. (d) Cryo-X-ray ptychography and 3D color rendering of myelinated axons in mouse brain tissue. Left panel is a single orthoslice from a 3D reconstruction (right). Nuclei (yellow); myelinated axons (blue); spherical structures (pink). Adapted with permission from ref (71). Copyright 2017 Springer Nature. (e) XRF mapping of MoS_2_ NPs in liver and spleen, showing the localization in liver sinusoid and splenic red pulp. Adapted with permission from ref (30). Copyright 2021 Springer Nature.

X-ray microscopy is a useful tool to provide detailed 2D and 3D information about tissue morphology and distribution of NPs from whole organ to subcellular levels (Figure 7).^30,71,96−99^ X-ray microtomography has been used to image the whole neurons in mouse brain without tissue slicing or clearing, enabling the 3D investigation of brain cortical neurons at the cellular level with a micron/submicron resolution (Figure 7b).^96^ Dense neuronal networks including cortical pyramidal cells, various neurons and motor axons in *Drosophila melanogaster* (Figure 7c), and mouse nervous tissue were reconstructed by X-ray holographic nanotomography with sub-100 nm resolution.^97^ Cryo-X-ray ptychography allowed the imaging of myelinated axons and subcellular structures (density, size, and localization of the nuclei, lysosomal lipofuscin, and neuronal pigmented autophagic vacuoles) in mice brains at a spatial resolution of ∼100 nm (Figure 7d).^71^ The distinction of the tissue architecture is fundamental and critical to confirming the location of nanomedicines and understanding the nano–bio interactions. Although there are no application examples, these imaging techniques have great potential to investigate the *in-vivo* distribution of nanomedicines.

So far, X-ray fluorescence (XRF) imaging has been widely used to investigate the location of nanomaterials in various tissues and model organisms.^30,100−104^ Chen and coauthors investigated the biodistribution of several NPs (MoS_2_, Au@Gd, Cu, etc.) in different mammalian organs and *Caenorhabditis elegans* (*C. elegans*) with hard XRF.^30,102−104^ The authors demonstrated that MoS_2_ was mainly present in liver sinusoids and splenic red pulp after intravenous injection (Figure 7e).^30^ Additionally, the effects of nanomedicine exposure on other biological elements can be analyzed since the fluorescence signals of multiple elements can be collected simultaneously. After intranasal instillation of Cu NPs, the amounts and distribution patterns of Fe, Ca, and Zn in substructures of the mouse brain changed dramatically.^103^ At the animal level, the combination of nano-CT and nano-XRF were used to map the Co NPs in *C. elegans* at the high resolution of 40–100 nm in 2D and 3D.^104^ The applications mentioned above are snapshots during the nanobio interactions. ALS can also provide advanced X-rays and great opportunities to dynamically investigate the interactions in cells and tissues.

### 3.5 Dynamic Tracking of Nano–bio Interactions in Live Cells and Animals by ALS Microscopy

The nano–bio interaction is usually a dynamic process; the biodistribution of nanomaterials in tissues or cells, as an example, is a rapid delivery/transport action immediately after administration. Time-lapse recording of the process is tantalizing for researchers to understand the animated changes of nanomedicines and biological structures. ALS X-rays with high flux and coherence show great advantages in dynamic imaging over conventional X-ray sources. Yet, it is complex and difficult to develop the live cell or animal-adapted system in the beamline since ALS facilities are not designed specifically for living biological samples. Therefore, most of the ALS-based studies have focused on fixed and dead cells or tissues. So far, real-time imaging of living cells and animals by ALS techniques is in the initial stage of development. The key challenge is the radiation damage caused by X-rays with high flux and intensity. In living animal imaging, the radiation damage can be reduced by minimizing the total dose, which nevertheless limits the desired resolution. The development course of a living embryo was examined by time-lapse X-ray microtomography.^105^ The respiratory structure,^106^ the liquid/particulate instillations,^107,108^ and lung biomechanics^109^ in living animals (mice, rats, pigs) were visualized with phase-contrast X-ray imaging. The delivery and distribution process of Au NPs in lung after venous injection were investigated by X-ray microradiology.^107^ For the imaging of live cells, an X-ray-free electron laser (XFEL) with femtosecond pulse and extremely high coherence and brilliance can overcome radiation damage by “freezing” samples in a femtosecond time period and imaging with the “diffraction-before-destruction” principle. CDI with an XFEL source has great potential to visualize nano–bio interactions in live cells with nanometric resolution, which has successfully imaged mimivirus,^110^ live bacteria,^111,112^ and Au nanoclusters-labeled live bacteria.^113^ We believe ALS microscopy will be a powerful tool to track the nano–bio interactions *in vitro* and *in vivo* dynamically.

## 4 Examination of the Biotransformation of Nanomedicines by X-ray Spectroscopy Techniques

**Figure 8:**
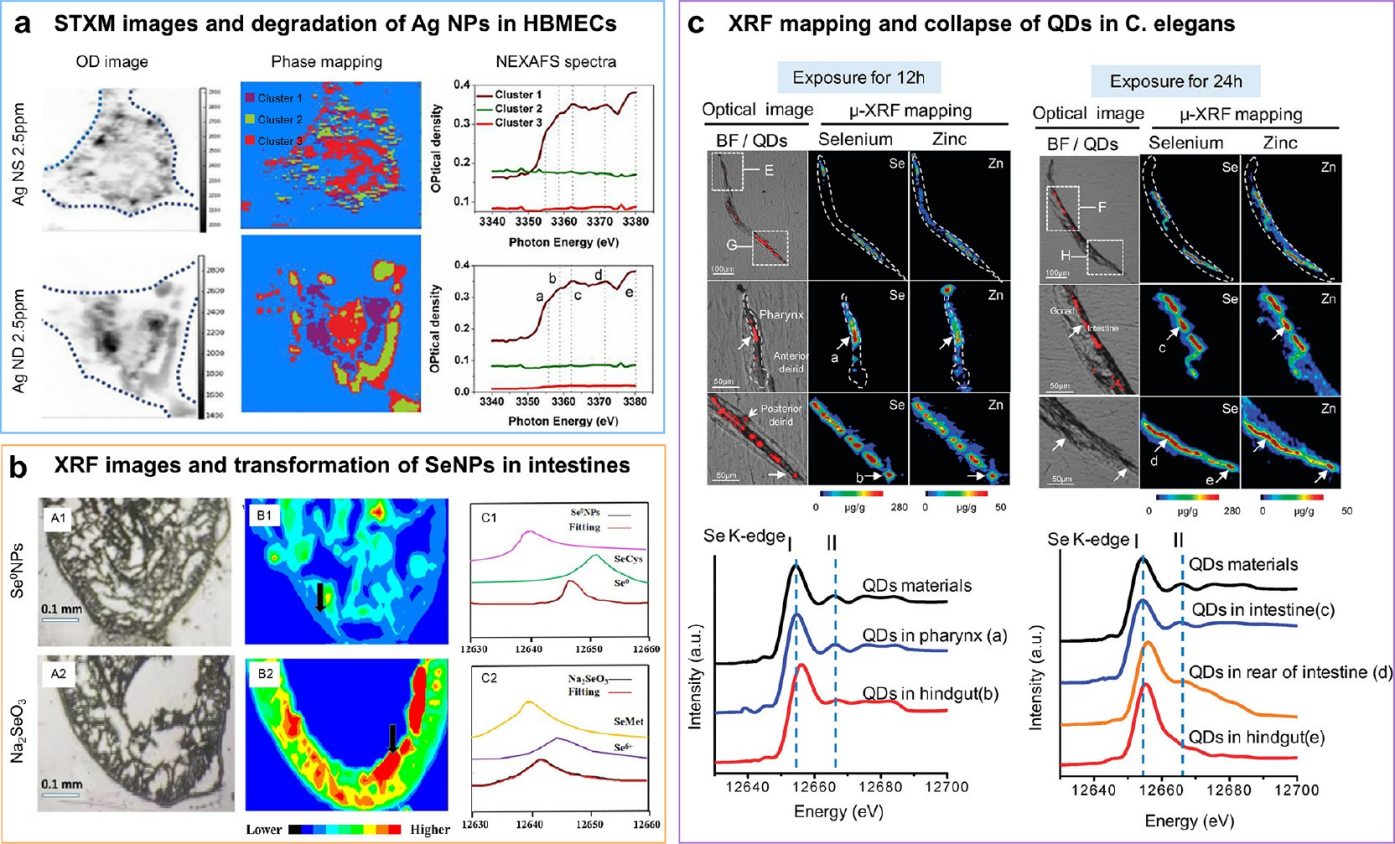
Investigation of the biodistribution and biotransformation of NPs in tissues and organisms. (a) STXM images of Ag NS and Ag ND in HBMECs of the BBB and Ag L-edge NEXAFS of three clusters (different compositions obtained by cluster analysis) in STXM images (Cluster 1 was identified as an Ag species). Reproduced with permission from ref (78). Copyright 2021 National Academy of Science. (b) The spatial distribution and chemical forms of Se in murine small intestine were determined by micro-XRF imaging and *in-situ* XANES at the position indicated by the black arrows. Reproduced with permission from ref (115). Copyright 2021 Elsevier. (c) Biodistribution and collapse of CdSe@ZnS QDs in *C. elegans*, as revealed by μ-XRF imaging (upper panel) and the corresponding XANES (lower panel) at the positions displayed by the white arrows labeled a–e. Adapted with permission from ref (104). Copyright 2011 American Chemical Society.

The stabilities of nanomedicines before and after exerting their functions determine their medical efficacy and safety. The interactions of nanomedicines with diverse biochemical factors, such as oxidoreductase enzymes, acidity, oxidants, to name a few, can induce the transformation of NPs. With the XANES technique, the acidic lysosomal environment, oxygen, cysteine, and tissue specific biomolecules have been demonstrated to contribute to the degradation, transformation, clearance, and bioavailability of nanomedicines.^28,30,114^ A popular method combining ALS imaging and XAS from the cellular to the organismal levels provides structure and morphology information, as well as the distribution and chemical forms of nanomedicines *in situ*.^78,104,115,116^ Valsami-Jones et al. examined the impacts of the biotransformation of metallic nanomaterials on the transport behavior through the blood–brain barrier (BBB).^78^ The authors confirmed a higher degradation rate of Ag nanodisks (NDs) than that of Ag NSs in human primary brain microvascular endothelial cells (HBMECs) using STXM phase imaging and *in-situ* XAS (Figure 8a), which, in turn, stimulated the transport of Ag NDs through the BBB. Li et al. compared the *in-vivo* behavior of SeNPs with Na_2_SeO_3_ in the small intestine by XRF mapping and *in-situ* XAS,^115^ finding a lower intake and lower toxicity of SeNPs (Figure 8b). SeNPs were mainly transformed to selenocysteine, while the chemical forms were selenomethionine and Se^6+^ in the Na_2_SeO_3_ group. At the organismal level, the *in-situ* biodistribution and degradation of CdSe@ZnS quantum dots (QDs) within the digestive tract of *C. elegans* were assessed via XAS together with XRF imaging (Figure 8c),^104^ demonstrating the decomposition of the core–shell nanostructure and the oxidation of Se^2–^ to SeO_3_^2–^ in the NP core. Compared with fluorescence microscopy, ALS imaging techniques provide more accurate information on partially degraded or fluorescence-quenched nanomaterials.

## 5 Summary and Outlook

**Figure 9:**
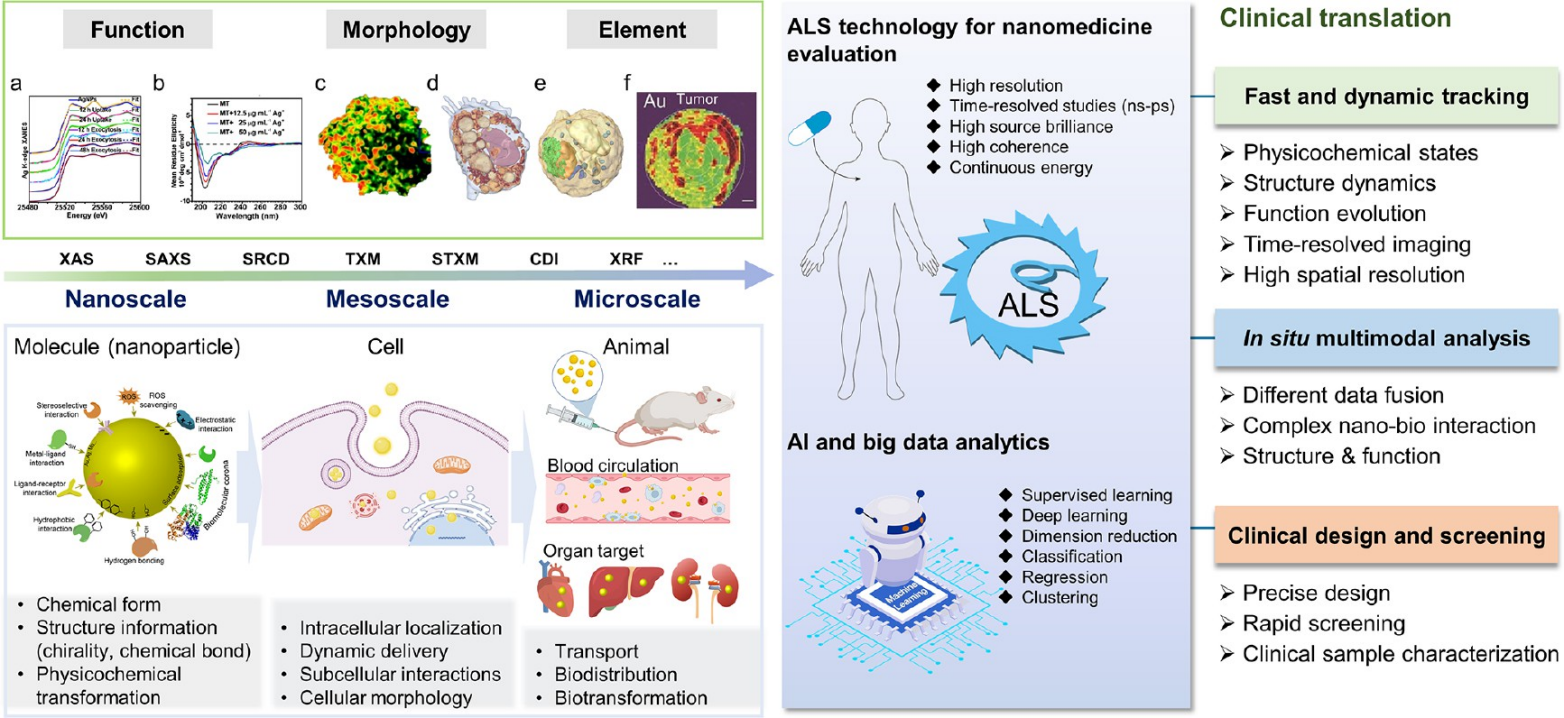
Summary of the current ALS technologies used to explore the biological behavior and fate of nanomedicines and insights for future development. Panels a–c, d, e, and f are adapted with permission from refs (28, 77, 68, and 102) respectively. Copyright 2015 American Chemical Society, Published in 2018 under a Creative Commons license, Copyright 2010 National Academy of Sciences, and Copyright 2016 Wiley, respectively. Images of cell and animal in bottom left panel are created with BioRender.com.

The ALS analytical methods have found numerous interesting applications in nanomedicine research, in view of the *in-situ*, nondestructive, high resolution, and element-specific properties of these techniques. At present, available X-ray microscopes and spectroscopy can provide independent or absorption-based spectroscopic information or 3D structure information at the nanometric resolution. These techniques have been important in the *in-situ* tomography of cell and organelle morphology, as well as the distribution, aggregation, and transformation of nanomedicines at the animal, cell, and subcellular levels. Herein, we discussed several, major SR-based X-ray imaging and spectroscopy techniques (summarized in Table 2 and Figure 9), focusing on the working principles and providing some prime examples of their applications.

**Table 2:** ALS-Based Imaging and XAS Techniques Used to Analyze the Biological Behavior and Fate of Nanomaterials

X-ray techniques	dimensions	analysis of tissue, cellular or sub- cellular structures	biodistribution of NPs in biological samples	chemical forms	examples
soft X-ray TXM and STXM	2D/3D	• Unstained samples	• In-situ imaging NPs in a single cell	no report	• Internalization of MoS_2_ NPs in blood cells^30^
		• A single cell, subcellular structure			• Organization of insulin vesicles and cytosol variations in intact β cells^70^
					• La@GO NPs in *E. coli*(74)
					• Gd@C_82_(OH)_22_ NPs in macrophages^77^
					• Formation of organic NPs in HeLa cells^75^
					• HeLa cells interacted with Fe_3_O_4_–SiO_2_ core–shell NPs^79^
					• Organelles in A549 cells incubated with Au NPs^89^
					• Imaging of intracellular proteins^93,94^
hard X-ray TXM	2D/3D	• Unstained samples	• In-situ imaging NPs in a single cell, multiple cells or tissues	no report	• Internalization of Ag NPs by THP-1 cells^28^
		• Thicker cells, tissues, organism			• MnARK in DC cells^91^
					• HeLa cells incubated with TiO_2_ NPs^90^
					• MoS_2_ nanosheets in a single hepatoma cell^92^
					• Elemental mapping of Co NPs in *C. elegans*(101)
					• 3D distribution of Ba-labeled macrophage in mice lung^99^
CDI	2D/3D	• Unstained samples	• In-situ imaging of NPs in cells with higher resolution and contrast image	no report	• Imaging of whole yeast spore^68^
		• Thicker cells, subcellular structure, tissues			• Myelinated axons in mouse brain tissue^71^
		• Higher resolution and lower radiation dose			• HeLa cells with Fe_3_O_4_–SiO_2_ core–shell NPs^79^
					• Au NPs and organelles in unstained mouse breast cancer cells^85^
XRF	2D/3D	• Unstained samples	• Imaging NPs via element- specific fluorescence signal	combined with XANES	• MoS_2_@HSA in mice liver and spleen^30^
		• Organisms, tissues, cells, or subcellular structure	• Allowing multiple elements detection simultaneously		• Imaging OmpA proteins with lanthanide metal probes^95^
					• Cu NPs in mice brain^103^
					• Au@Gd NPs in tumors^102^
					• QDs and Co NPs in *C. elegans*(101,104)
					• Cu-complexes within *Drosophila melanogaster* via XANES tomography^100^
					• 3D elemental microtomography of *Cyclotella meneghiniana*(121)
					• Elemental mapping of Zn and K in PC 12 cells^122^
XAS	2D	• Unstained samples	combined with STXM imaging	quantifying chemical valence states and forms of NPs present in biological samples	• Oxidation of MoS_2_ NPs in the liver and spleen^30^
		• Chemical structures of elements in organisms, tissues, and cells			• Degradation of QDs in *C. elegans*(104)
					• Intracellular dissolution of Ag NPs^78^
					• Transformation of SeNPs in rat’s small intestine^115^

Over the lengthy development of nanotechnology research, nanomedicine has gradually moved toward the stage of clinical application. The precise design, rapid screening, risk prediction, and regulatory demands are significantly limited by the lack of accurate, *in-situ* characterization of the physicochemical properties of nanomedicines, quality control, manufacturing, and clinical evaluation. Although X-ray-based analytical techniques have led to tremendous inroads toward understanding the biological behavior and fate of nanomedicines, future technological development is still necessary to advance the application of these techniques in this area of research. The improved performance of globally accessible radiation sources, including third-generation synchrotron radiation (ESRF, SSRF, ALS, CLS, etc.), XFEL, and High Energy Photon Source (HEPS), under construction in China, are expected to provide more advanced X-ray sources with ultrahigh brilliance (exceeding 10^22^ photons s^–1^ mm^–2^ mrad^–2^), intensity, coherence, and repetition rates (3520 measured images per second) to provide super high resolution (nm) and time-resolved (ns–ps) analysis. It is also likely that greater feasibility with organic and inorganic nanomaterials will be realized.

It is increasingly important to develop superior analytical technologies based on next-generation ALS, with its markedly higher spatial and temporal resolution, multimodal data fusion, and intelligent prediction abilities, to fully understand the currently enigmatic features of nanomedicines.

(1) At the molecular level, the nanobio interaction is usually a rapid and dynamic process; suitable beamlines combined with XFEL may be promising methods for the ultrafast and dynamic tracking of physicochemical states, structure dynamics, function evolution, time-resolved (ps–ns), and high spatial resolution imaging, down to the atomic scale.

(2) At the cellular and animal level, nanobio interactions usually exhibit multiple complex processes and participants, requiring multimodal analysis to provide comprehensive information involving structures, elements, functions, etc. The ideal strategy is to develop *in-situ* and correlative multimodal instruments at a single ALS end station that combines different bioanalysis apparatus, such as super-resolution fluorescence microscopy, electron microscopy, and mass spectrometry, with different X-ray microscopes (XRF, TXM, CDI, STXM, X-ray ptychography, X-ray holography, etc.). Light- and electron-based microscopy offer structural and cellular information to buttress the ALS data, while mass spectroscopy provides molecular context. Thus, full and complementary information from the same sample can be displayed. Currently, the multimodal correlative ALS microscope and algorithms in one single synchrotron end station have become one of the mainstream trends in worldwide light source beamline development.^83,87,117−120^

We believe that, in the near future, both the major advancement of next-generation ALS and the development of corresponding integrated device control systems and algorithms will facilitate the efficient collection and analysis of different types of data in both speed and accuracy, improving quantitative downstream image analysis with exceptional three-dimensional resolution. The advantage of these microscopes will enable the simultaneous collection of big data from various measurement modes, such as absorption, scattering, fluorescence, etc., providing an in-depth analysis of complex nanobio interaction processes and their correlation with consequent changes in biological activity.

(3) The higher temporospatial resolution or multimodal analysis can increase the radiation dose and introduce the risk of radiation damage to biological samples. Thus, it is necessary to develop cryogenic sample environments, optimized data acquisition processes, and efficient control algorithms to ensure the collection of accurate and faithful information. Simultaneous data acquisition is preferred since sequential analysis introduces more radiation and damage to biological specimens. Additionally, the movement of samples in sequential imaging makes data reconstruction difficult. Moreover, the analysis time of data simultaneously obtained can be reduced because the data set used for image alignment/segmentation in one method can be directly applied to the other imaging modality.

(4) All data collection and processing, including different, large data sets (absorption, scattering, fluorescence, etc.), imaging data correlation, and fusion and segmentation processing, will increase the dimension and complexity and eventually provide more comprehensive nanobio interaction information. The upgrades to more advanced light sources and the improved big data analysis algorithms (e.g., machine learning) should be developed to reduce the signal noise and time cost and increase reconstruction precision. The data fusion process is the simple superposition of respective modal images and makes full use of the complementarity of each technique’s information, thereby exploiting the advantages of respective modal images, providing more powerful and abundant information for specific research applications, and expanding the applications of ALS analytical techniques in the field of nanomedicine.

(5) To promote the clinical translation of nanomedicines, the ALS sample preparation procedure and data acquisition methods should be improved to increase the design precision, rapid screening, and clinical sample characterization, all of which would enable more robust and accurate evaluations of nanomedicines.

In summary, this Outlook provides a review on the current state of ALS in nanomedicine and infers that the collaboration of scientists, ALS beamline engineers, and clinicians may form a positive-feedback loop that ultimately leads to the clinical translation of nanomedicines. We look forward to the next generation of ALS analysis in which the frontiers of XFEL techniques expand with the help of new X-ray nanoprobes, artificial intelligence, and machine learning. We hope this Outlook will inspire new research endeavors that expand the potential and application of these techniques.
